# Cortical circuits modulate mouse social vocalizations

**DOI:** 10.1126/sciadv.ade6992

**Published:** 2023-09-29

**Authors:** Benjamin Gan-Or, Michael London

**Affiliations:** Edmond and Lily Safra Center for Brain Sciences and Alexander Silberman Institute of Life Sciences, The Hebrew University of Jerusalem, Jerusalem 91904, Israel.

## Abstract

Vocalizations provide a means of communication with high fidelity and information rate for many species. Diencephalon and brainstem neural circuits have been shown to control mouse vocal production; however, the role of cortical circuits in this process is debatable. Using electrical and optogenetic stimulation, we identified a cortical region in the anterior cingulate cortex in which stimulation elicits ultrasonic vocalizations. Moreover, fiber photometry showed an increase in Ca^2+^ dynamics preceding vocal initiation, whereas optogenetic suppression in this cortical area caused mice to emit fewer vocalizations. Last, electrophysiological recordings indicated a differential increase in neural activity in response to female social exposure dependent on vocal output. Together, these results indicate that the cortex is a key node in the neuronal circuits controlling vocal behavior in mice.

## Teaser

The production of mouse social vocalizations directly involves higher-level brain activity.

## INTRODUCTION

Humans and a wide variety of animals use the vocal production of sound to facilitate a high-throughput and high-fidelity means of communication that does not require visual or physical contact. Mice emit ultrasonic vocalizations (USVs) in various contexts (*1*–*4*) with one prominent instance being during courtship behavior, in which mice emit simple and complex USVs (*5*–*7*) (movie S1). Most of the evidence indicates that laboratory mouse USV emission is an innate behavior with little or no role for learning (*8*–*10*). Neural mechanisms in the subcortical forebrain and midbrain that directly control USV behavior were recently illuminated. It has been shown that neurons in the preoptic area (POA), periaqueductal gray (PAG), nucleus ambiguus, and nucleus solitary tract are necessary and sufficient for USV emission and their modulation controls specific USV properties such as bout length or syllable amplitude (*11*–*16*). Naturally emitted USV bouts show a substantial level of complexity (*11*–*13*, *17*–*19*). At present, it is unclear whether POA- and PAG-induced USV bouts show the same degree of complexity. Other neural circuits, specifically cortical circuits and their projections, may contribute to the complexity of this behavior.

While in mammals such as humans, monkeys, and bats, there are cortical circuits dedicated to vocalization (*20*, *21*), the involvement of the cortex in the production and control of USVs in mice is subject to debate. Arriaga *et al.* (*22*) reported a direct projection from the primary motor cortex to laryngeal motoneurons. They also found that lesioning M1 subtly modulates the frequency component of USV repertoire but does not affect syllable composition or amplitude. In wild neotropical singing mice, the cortex was required to maintain their natural vocal turn-taking behavior (*23*). In sharp contrast, using genetic ablation of the entire population of excitatory neurons in the cerebral cortex, Hammerschmidt *et al.* (*24*) have shown that the cortex is not necessary for USV production in mice and that these mice lacking cortex from birth produce USVs indistinguishable from control mice. However, later analysis of these identical data showed that artificial deep neural networks could be trained to distinguish control mouse USVs from those emitted by mice lacking cortex (*25*). Studies in mammals demonstrating the involvement of cortical circuits in vocalizations point to the anterior cingulate cortex (ACC) as a key cortical area. For example, intracortical electrical microstimulation (ICMS) of the ACC evokes vocalization in several mammals including rats, bats, guinea pigs, squirrel monkeys, macaques, and humans (*26*–*31*). Mice have a homolog of some regions of the human ACC (*32*); however, no study examined the ACC in relation to mouse USVs. Furthermore, to our knowledge, no study has examined functional cortical neural activity in the mouse during USV production.

The evidence reviewed so far sets specific expectations for a possible role for cortical circuits in controlling USV emission. The sufficiency of subcortical circuits in USV production combined with the strong inhibition involved in this process indicates that a candidate cortical circuit is likely to play a modulatory role in the behavior. Hence, it is not anticipated that manipulating cortical circuits will robustly initiate or abolish USVs in comparison to manipulating subcortical circuits. Rather, cortical activity is expected to change in certain aspects before and in accordance with USV production.

To address this question of a cortical role in mouse USV production, we have performed ICMS, optogenetic stimulation, fiber-photometry recordings, and optogenetic silencing of ACC. To further elucidate the neural activity correlate, we used electrophysiology in this region in vocalizing head-restrained mice to determine the specific cortical activity preceding USV production. We conclude that a subregion of the mouse ACC is involved in the modulation of USV behavior during courtship.

## RESULTS

### Intracortical microstimulation in ACC triggers USV

**Fig. 1.**
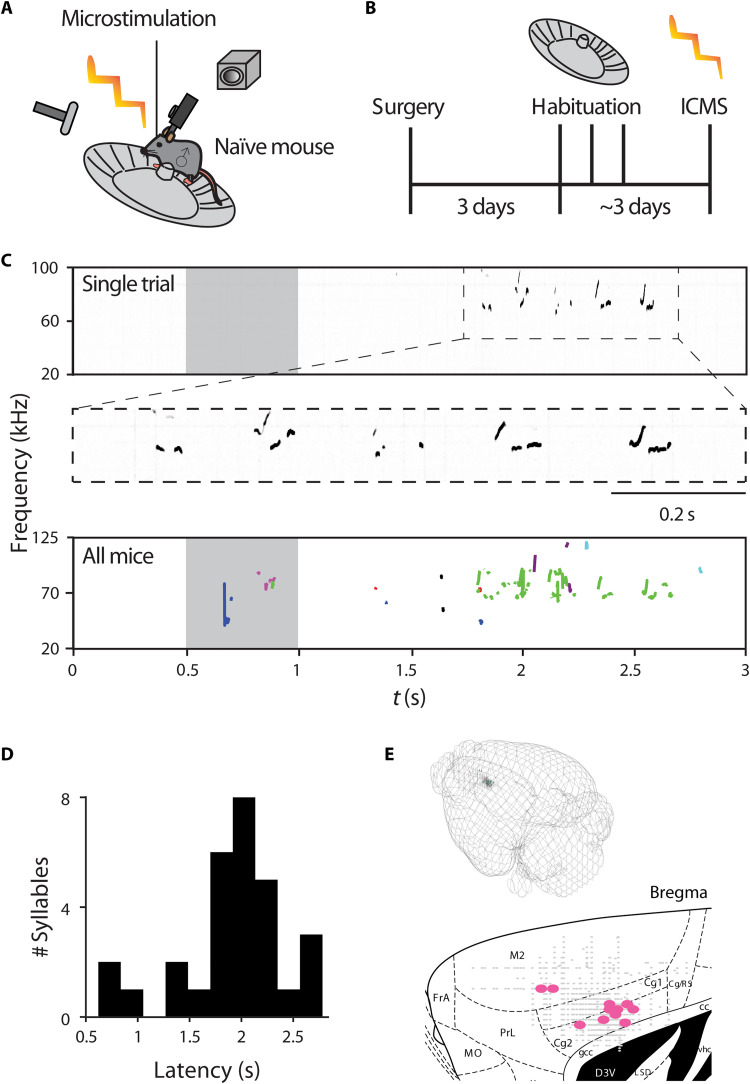
Microstimulation in a specific location in ACC induces USV. (**A**) Schematic illustration of the experimental setup. A glass pipette is used to induce microstimulation in the cortex of a head-restrained male mouse positioned on a running wheel, while an ultrasonic microphone records USVs and a camera records any behavioral response. Male mice are naïve to female exposure from weaning at P21. (**B**) Schematic illustration of experimental preparation. After head restraint, mice recover for 3 days and subsequently undergo habituation to the head-restraint apparatus lasting about 3 days. (**C**) A single stimulation trial spectrogram example of USVs elicited by the mouse following ACC microstimulation (stimulus duration of 500 ms). Below is a zoomed-in spectrogram showing that the evoked sound is complex and in the ultrasonic range. Further below is an overlay of all USVs (*n* = 29 syllables) induced in all mice with each color representing a different mouse (*n* = 8 mice). (**D**) Histogram of latency from stimulation onset to USV syllable. (**E**) A 3D visualization of all stimulation sites, with USV-inducing sites in green and quiet sites in gray. Below is a sagittal section with coordinates of locations tested by ICMS (gray markers denote locations that did not induce any USVs, while pink markers denote locations where at least one USV was induced).

In view of the seemingly contradictory evidence for the involvement of the cortex in the control of USVs in mice (*8*, *24*), we have tested whether electrical activation of the mouse’s cortex can trigger USVs. Previous studies have demonstrated that ICMS in the cortex of various other mammals triggers vocalization (*23*, *26*, *29*, *33*). Using ICMS, we performed a systematic mapping of the motor and premotor areas to determine whether USVs can be induced in male mice. Male mice only emit USVs while awake; therefore, a systematic large-scale mapping of possible vocal nodes requires head restraint. Male mice naïve to female exposure were habituated to head restraint (Fig. 1A and movie S2). To prevent any hint of female scent in the ICMS experiments, we never exposed mice to females on the head-restraint apparatus and never brought females into the rig throughout the habituation procedure (Fig. 1B). No mice produced any detectable spontaneous USVs while head restrained [*n* = 23 mice, total recording time = 61 hours; see also (*34*)]. This complete absence of spontaneous USVs provided an ideal setting to test for USVs triggered by ICMS.

We then performed penetrations over the cortical area at various depths at each location (advancing in vertical steps of 50 μm). Following the work of Graziano *et al.* (*35*) in which long-duration ICMS resulted in complex naturalistic movements in monkeys, we have used at each location both short- and long-duration stimulation trains (short, 50 ms; long, 200 to 500 ms; Materials and Methods). In five mice, short- and long-duration ICMS in the primary motor cortex (M1) and secondary motor cortex (M2) produced a large variety of simple (twitches) and complex movements; see also (*36*)] but have not resulted in any USV production (# USVs = 0, 1148 penetrations, 11,203 stimulations). In addition, we have stimulated at the coordinates reported in a previous report (*8*) within M1 but failed to induce USVs. Then, we set out to test ICMS in the cingulate cortex (ACC) (*37*), and in sharp contrast, at a clustered location in the anterior ventral ACC, long-duration ICMS evoked USVs (bregma: 700 ± 100 μm medial, 800 ± 300 μm anterior, 1500 ± 300–μm depth; Fig. 1E). An example of an individual USV and several USVs in succession following a 500-ms-long ICMS is depicted in Fig. 1C and movie S3. These vocalizations were in the ultrasonic range and did not represent stress-related sonic vocalizations (*38*). An overlay of all ICMS-induced USVs in successful stimulation sites across all mice is shown in Fig. 1C (total ICMS sites = 1974, successful sites = 12, number of triggered USVs = 29, *n* = 8 mice). Only long-duration stimulations resulted in USVs, while short-duration ICMS at the same locations did not induce USVs. Long-duration ICMS-induced USVs had an average frequency of 81 kHz and varied in duration (see full statistics in table S1). USVs were only triggered by long-duration ICMS with relatively long latencies to USV emission (average latency to first USV: 1.5 ± 0.7 s, *N* = 8; Fig. 1D), suggesting a complex involvement of ACC in USV production. This response latency is in agreement with ICMS-induced USVs in the ACC of monkeys and rats (*26*, *39*). In conclusion, we have identified a location within the ACC of male mice where long-duration ICMS evokes the initiation of USVs, albeit with a low success rate and long latency.

### Bilateral optogenetic stimulation of pyramidal neurons in ACC elicits sequences of USVs

**Fig. 2.**
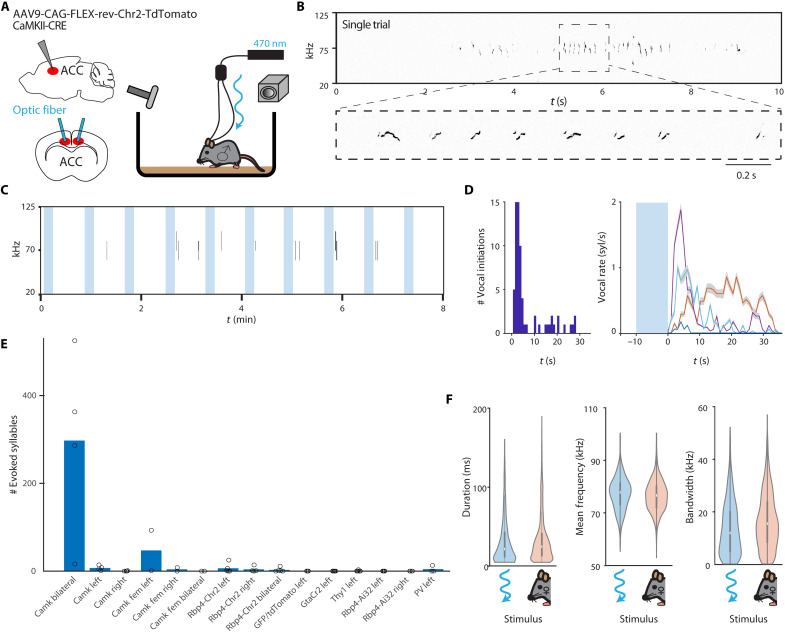
Optogenetic stimulation in ACC induces sequences of USVs. (**A**) Schematic illustration of the optogenetic activation setup. A male mouse with bilateral optical fiber implants and injected Chr2 excitatory vector freely moves in a cage (in solitude). (**B**) An example spectrogram following a single bilateral ACC optogenetic stimulation showing a sequence of USVs elicited (stimulus immediately precedes start of example). Below is a zoomed-in spectrogram; note that these vocalizations are similar to the normal mouse vocal repertoire. (**C**) An example session of 8 min with 10 stimuli (laser intensity = 1.5 to 3 mW/mm^2^), each lasting 10 s with ~30 s of rest between stimuli. Vocalizations can be seen as long black lines representing the start times and frequency range. (**D**) Left: A histogram of latency from stimulation offset to USV onset. Right: A plot of syllable rate by mouse with shaded area representing SD showing that most USVs occurred about 10 s after stimulation offset (*N* = 67 successful stimuli total). (**E**) Comparing number of evoked syllables across animals across all tested optogenetic stimulation experiments. All animals are represented as circles. (**F**) Comparing syllable features between animals from stimulation-induced and female-induced USVs. From left to right is duration (*P* = 0.27), mean frequency (*P* = 0.75), and bandwidth (*P* = 0.50) comparisons. In all of the features, the distribution is highly similar between USVs evoked from optogenetic stimulation and female presentation.

ICMS invoked short sequences of syllables, nonrobustly and with low spatial specificity. Moreover, ICMS-induced USVs could result from stimulation of passing axons rather than ACC neurons (*40*). Therefore, we have tested whether optogenetic activation of neurons in the same location in ACC triggers USVs. In the PAG and other subcortical structures, optogenetic stimulation reliably evoked vocalizations (*11*–*13*). We expressed Cre-dependent ChR2 in mouse calcium- and calmodulin-dependent protein kinase II–positive (CamkII–positive) neurons bilaterally in the ACC and implanted optical fibers above the injection point [diameter = 200 μm, numerical aperture (NA) = 0.22] in the coordinates specified above. Two weeks after injection, we used light stimulation (trains of 10-ms pulses at 10 Hz for 10 s at laser intensity 1.5 to 3 mW/mm^2^) while mice were exploring a clean cage. Bilateral stimulation elicited full USV sequences (example post-stimulus vocal behavior and zoomed-in vocal sequence in Fig. 2B and movie S4, example session in Fig. 2C). Mice never vocalized during the optogenetic stimulation and elicited vocalizations occurred at a minimum of 0.95 s after stimulation with an average of 8-s delay (Fig. 2D). Typically, USVs were emitted in the first 10 s after stimulation (Fig. 2D).

We also performed optogenetic stimulation across various conditions and strains including in females. We found that only bilateral stimulation in the CamkII-Cre males elicited robust vocal behavior (Fig. 2E; *N* = 67 successful stimuli in four mice, *N* = 1190 syllables). Unilateral stimulation occasionally resulted in a single short USV but with a low success rate. In mouse strains where expression was limited only to layer 5 pyramidal neurons (Thy1-ChR2 and RBP4-Cre), we failed to reliably trigger USVs. As opposed to ICMS-induced vocalizations and unilateral optogenetic stimulation–induced vocalizations, bilateral CamkII stimulation–induced USVs were frequent enough so that they could be compared statistically to female-induced USVs (Fig 2F). Stimulation-induced USVs had an average frequency of 77.5 ± 0.4 kHz compared to 77.2 ± 1.1 kHz in female-induced USVs in the same mice (*n* = 4 mice, *P* = 0.75, paired *t* test). In terms of duration, stimulus-induced USVs averaged 21.4 ± 7.5 ms, while female-induced USVs averaged at 27.1 ± 6.8 ms (*n* = 4 mice, *P* = 0.27, paired *t* test). The frequency bandwidth of stimulus-induced USVs was 11.7 ± 1.9 kHz, while the female-induced USVs were 13 ± 3.4 kHz (*n* = 4 mice, *P* = 0.50, paired *t* test). In summary, ACC bilateral ontogenetic activation in CamkII-positive male mice (in the absence of a female) robustly induced full sequences of USV that were not significantly different in their acoustic properties from natural courtship USVs in the same mice.

### Increased neural activity in the ACC precedes vocal onset

**Fig. 3.**
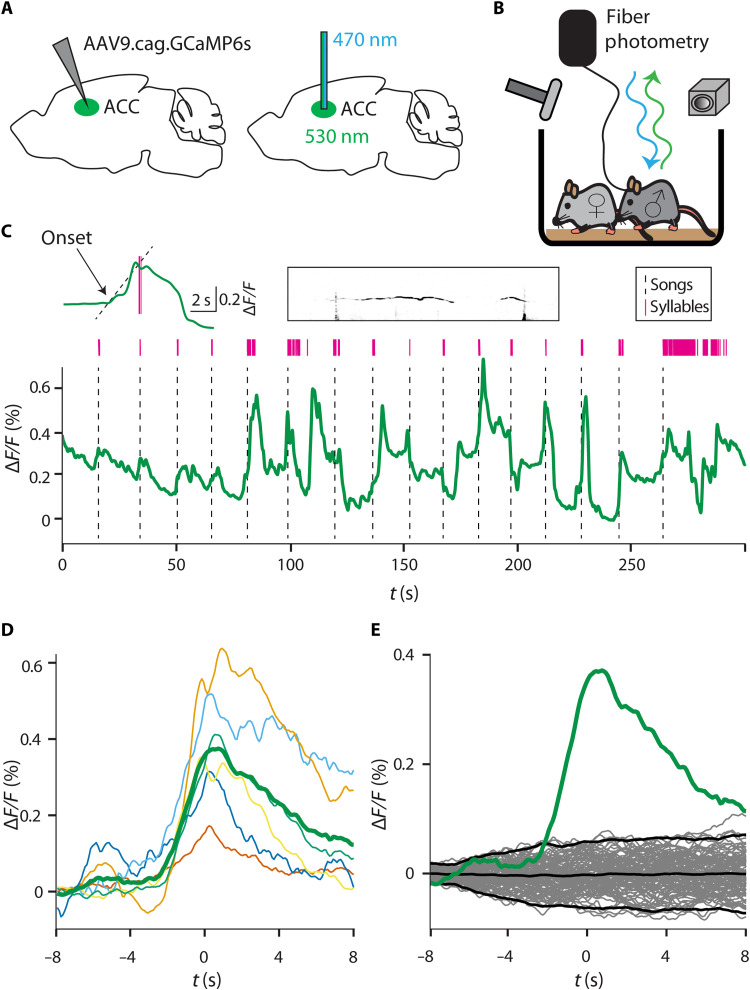
ACC Ca^2+^ activity correlates with song initiation. (**A**) Virus with Ca^2+^ indicator was injected 2 to 3 weeks before recording. Recordings were made from the left ACC using blue light to activate the Ca^2+^ indicator, and green light was detected in the photodiode. (**B**) Schematic illustration of fiber-photometry setup. Male mice with fiber implants were allowed to move freely in a cage, while a female was introduced to elicit USVs. (**C**) Example session of 5 min in duration. The green line represents Ca^2+^ signal, thin magenta lines are syllables, and the dotted black line represents song initiations. Above is zoom-in on a single song (with a dotted line showing activity onset calculation), with the spectrogram to the right showing the first two syllables in the song. (**D**) Average Ca^2+^ signals for all mice, triggered on the first syllable in a song separated by at least 10 s. (**E**) Permutation test trials using random trial times plotted in gray. The average from (D) in green is plotted against the average and ±2 SD of the permutation test average in black.

To explore the activity of the ACC neuronal population during natural courtship USV behavior, we used fiber-photometry population Ca^2+^ imaging (*41*). We performed stereotactic injections of Adeno-associated virus (AAV) viral vectors encoding for Ca^2+^ indicator AAV9.cag.GCaMP6s (see Materials and Methods) (*42*) into the location identified using ICMS in the left ACC (Fig. 3A). After expression, an optical fiber (diameter = 200 μm, NA = 0.22) was implanted in the coordinates specified above, and we measured Ca^2+^ dynamics using fiber photometry in a natural courtship setting as depicted in Fig. 3B and movie S5 (see Materials and Methods). Mice implanted with an optical fiber were placed in a fresh cage for a short habituation period (5 min). Then, the session began, and for a duration of 20 min, a female mouse was repeatedly presented to induce USV production and then immediately removed from the cage. During the session, the Ca^2+^ signal and the microphone signal were recorded continuously. The male mice often responded to female entry by emitting USVs. On the basis of the distribution of the intersyllable interval, we defined a song as a succession of syllables with a surrounding silent period of at least 10 s of quiet (see Materials and Methods for syllable and song definitions) (*18*). Six mice were recorded for at least one session, totaling 33 hours of recording with 2176 songs (mean, 363 songs per mouse).

Ca^2+^ transients showed a clear temporal correlation with song initiation (Fig. 3C). Averaging the Ca^2+^ signal aligned on song initiation (the time of occurrence of the first syllable in every song) revealed an elevation in Ca^2+^ activity (Fig. 3D). To test whether this elevation in the Ca^2+^ signal is significant, we used a permutation test. We generated a sequence of random times to trigger the Ca^2+^ signal and calculated the distribution of the expected Ca^2+^ signal from the data (Fig. 3E). We then compared the actual signal to the expected one for each mouse. Five of six mice passed this test with *P* < 0.05. To control for a possible movement artifact, we also tested mice using the same procedure, but injected with a control green fluorescent protein (GFP) vector and found no increase in activity when triggering on USVs (fig. S1). To test whether the Ca^2+^ signal preceded or followed the song onset, we defined the Ca^2+^ onset by fitting a straight line to the range of 10 to 90% of its peak amplitude and extrapolated to zero crossing (see Fig. 3C, upper-left inset). We found that the Ca^2+^ elevation begins before song onset. The average latency from Ca^2+^ signal onset to song initiation was 2.3 ± 3.8 s (*n* = 6). This temporal difference is in good agreement with the delay reported in the stimulations experiment above (taking into account the slow dynamics of the Ca^2+^ signal and its low-pass filtering). These results demonstrate an elevation in activity in the same ACC location in which stimulations trigger USVs. This elevation occurs before song initiation while mice are engaged in natural courtship behavior, further indicating cortical involvement in this behavior.

### Silencing ACC neural activity reduces syllable production and song initiation

**Fig. 4.**
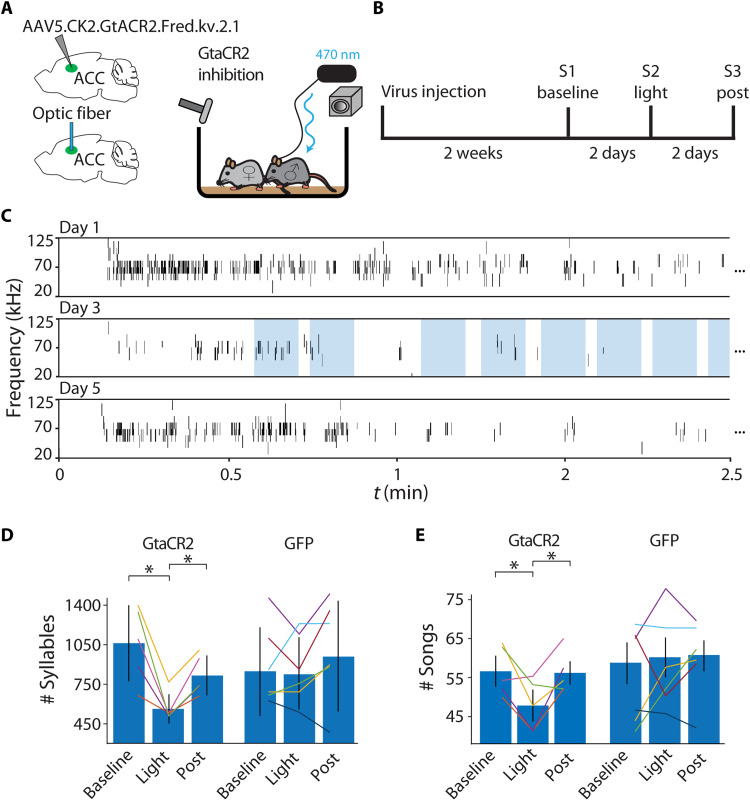
Suppressing ACC activity reduces emitted syllables and song initiation. (**A**) Schematic illustration of optogenetic suppression setup. A male mouse with optic fiber implant and injected GtACR2 inhibitory virus freely moves in a cage, while a female is placed by hand into the cage during experimental trials. (**B**) Schematic of the experimental timeline. Two weeks after virus injection, to allow for strong expression, session 1 baseline USV performance is assessed. After 2 days, session 2 USV emission is assessed with simultaneous optogenetic silencing. In session 3, after 2 days, post-stimulus USVs are assessed for recovery. (**C**) Example 2.5 min of USVs from a baseline session, stimulation session (light intensity = 1.5 to 3 mW/mm^2^), and post-stimulus session. Individual stimulation epochs are shown in light blue during the light session. (**D**) Mice emitted fewer syllables during ACC suppression. Percentage of syllables across the different sessions with average in blue bars with standard error. Control (GFP) sessions are compared on the right side. Baseline mice syllable emission is variable, but the stimulation session showed a significant decrease in comparison to the post-stimulus session 2 days after the stimulus session. (**E**) Mice emitted fewer songs during ACC suppression. The number of songs across the different sessions with average in blue bars with standard error. Control (GFP) sessions are compared on the right side. Compared to baseline, mice emitted fewer songs during the stimulation session and fully recovered in the post-stimulus session.

Previous studies have shown that activation of subcortical regions such as the PAG and POA is sufficient for triggering USVs and controlling some of their basic properties (such as syllable volume and bout duration) (*11*–*13*, *43*). Nevertheless, the effect of manipulation of cortical activity during courtship on vocalization has not been tested. To test whether silencing of the ACC would affect courtship USVs, we expressed the optogenetic silencing actuator *Guillardia theta* anion-conducting channelrhodopsin 2 (GtACR2) (*44*) unilaterally in the left hemisphere ACC of male mice targeted to the somata of pyramidal neuruons to obtain light-induced suppression of neural activity (Fig. 4A). After recovery from optic fiber implantation, mice were placed in a clear cage while connected to the laser without light stimulation while USVs were recorded continuously. The session started with a 5-min habituation without a female. Afterward, a female was introduced into the arena for an additional 20 min (experimental schematic in Fig. 4B). After 2 days, mice again met a female for 20 min with the laser-activated for 50 interspersed stimulation trains (duration = 10 s, frequency = 20 Hz, pulse duration = 10 ms, laser intensity = 1.5 to 3 mW/mm^2^). Example segments from the light sessions demonstrating eight stimulation trains are depicted in Fig. 4C and movie S6. The final post-stimulus session was conducted with the same behavioral protocol after 2 days. To test whether light silencing affected USVs, we compared the number of USVs emitted during light-delivery periods (blue sections in Fig. 4C) versus concurrent time intervals in the absence of light stimulation. The stimulation did not produce any observable disruption of normal courtship behavior, nor did it prevent vocal or motor behavior (fig. S2D). However, optogenetic suppression of ACC activity reduced the number of syllables produced, whereas the syllable main frequency component remained normal (Fig. 4C). Relative to baseline, the number of syllables emitted in the light delivery sessions was reduced and returned to baseline in the post-stimulus session (Fig. 4D; baseline, 1007 ± 174; suppressed, 343 ± 67; post, 681 ± 92; *P* < 0.05, *N* = 5). Mice also initiated fewer songs during ACC suppression compared to the baseline session while a recovery occurred in the post-stimulus session (Fig. 4E; baseline, 55 ± 3; suppressed, 48 ± 3; post, 57 ± 2; *P* < 0.05, *N* = 5). A basic analysis of temporal and spectral properties showed no vocal difference between the groups (fig. S2, A to C). A control group of mice injected with AAVdj.CMV.eGFP were tested using the same protocol. In the control group, no significant changes were found in the number of emitted syllables during stimulation trains or in song initiation (Fig. 4, D and E; no of syllables: baseline, 801 ± 159; suppressed, 767 ± 135; post, 964 ± 198; no. of songs: baseline, 54 ± 6; suppressed, 58 ± 5; post, 60 ± 4; *P* > 0.05, *N* = 6). The results of these experiments suggest that a reduction in ACC neural activity is associated with a reduction in syllable emission and song initiation, which further strengthens the conclusion that the ACC is an important node in the USV-controlling network.

### Differential responses of ACC and secondary motor cortex neurons to social exposure conditioned on song initiation

**Fig. 5.**
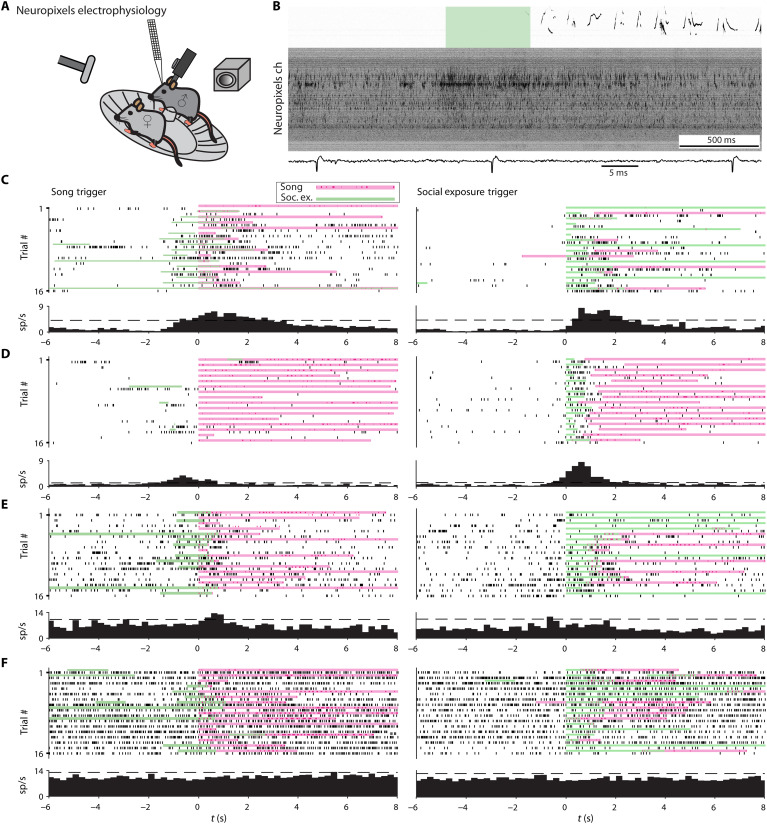
Both SE and song initiation modulate cortical single-unit activity. (**A**) Schematic illustration of the experimental setup. A Neuropixels probe is inserted into the ACC of a head-restrained male mouse (positioned on a running wheel), while an ultrasonic microphone records USVs and a camera records any behavioral response. (**B**) Example of the recording quality of all channels in response to SE and subsequent song initiation. SE epoch is in light green superimposed over the spectrogram of the recorded sound. Below is a zoomed-in example of a single channel. Only units passing 10 SDs above the mean waveform value are included in the analysis. (**C** to **F**) Example raster plots of single-unit activity. Each unit has two panels: triggered on a song (left) and triggered on SE (right). Magenta stripes denote song (ticks are the individual USVs), and green stripes mark the time and duration of SE. The left panels show 16 trials when the mouse was emitting USVs aligned on song onset (sometimes in response to SE and sometimes not). The trials on the right show neural activity while the male was given female exposure triggered on SE onset (sometimes the mouse emitted USVs and sometimes not). (C) This example shows a single unit responding both when triggered on the initiation of vocalization and when triggered on SE. (D) This unit responds weakly when triggered on song and more strongly when triggered on SE. (E) A unit with a weak response to song initiation and no response to SE. (F) An example of a single unit not responding to either song or SE.

The series of experiments described above provides one correlative and two independent causal lines of evidence showing that the ACC is involved in USV control. Despite this, the recorded signals and manipulations do not permit spatial and temporal resolution down to the level of individual neurons and spikes. We next wanted to explore the role of individual neurons in this region of the ACC during social USVs. Consequently, we performed electrophysiological recordings at a greater spatial and temporal resolution. Adult male mice were habituated to the head-restraint apparatus used in Fig. 1A. We then used a Neuropixels system [(*45*); see Materials and Methods] to record at a depth of 2.5 to 3 mm in the ACC and M2 (coordinates 700 μm anterior, 700 μm lateral). In contrast to the ICMS experiment where head-restrained males were never exposed to females before or during the experiment, here, male mice were head restrained and female mice were introduced to trigger USVs. USVs and video were simultaneously recorded (Fig. 5A). We use the term social exposure (SE) to denote the female presentation as the male was not always directly interacting with the female. Mice usually responded to SE by emitting songs (65% of female presentations resulted in songs in 1079 of 1661 presentations). However, in some cases, SE led to songs induced with long latency, at times where the female was no longer present (constituting 11% of total song production). We defined a song as above, emphasizing here that 10 s of silent period between songs ensures that activity presong is likely to represent spontaneous activity rather than part of a longer vocalization bout (see Materials and Methods for syllable and song definitions).

The electrophysiological recordings had a high signal-to-noise ratio, obviating the need for spike sorting and allowing us to focus on single-unit activity (Fig. 5B; see Materials and Methods). In total, we recorded 130 units in three mice. Figure 4 (C to F) shows example raster plots and perievent time histograms (PETHs) triggered on song initiation or SE. Each row shows a different single unit, whereas the left column are trials triggered on the song initiation and the right column are trials triggered on the SE. All trials appear, both SEs with and without vocal output and vocal output that did not directly follow SE. We found a variety of units responding mainly to SE (Fig. 5D), song initiation (Fig. 5E), both (Fig. 5C), or none (Fig. 5F).

PETHs for all 130 units show that the population of neurons responded both to SE and to song initiation (fig. S3A). The time course of the population PETH triggered on song initiation is in good agreement with the average Ca^2+^-signal response shown in Fig. 3E. In addition, we reanalyzed the photometry experiment using the same separation between SE and song initiation and found similar delayed responses to song as compared to SE (fig. S4, A to C).

**Fig. 6.**
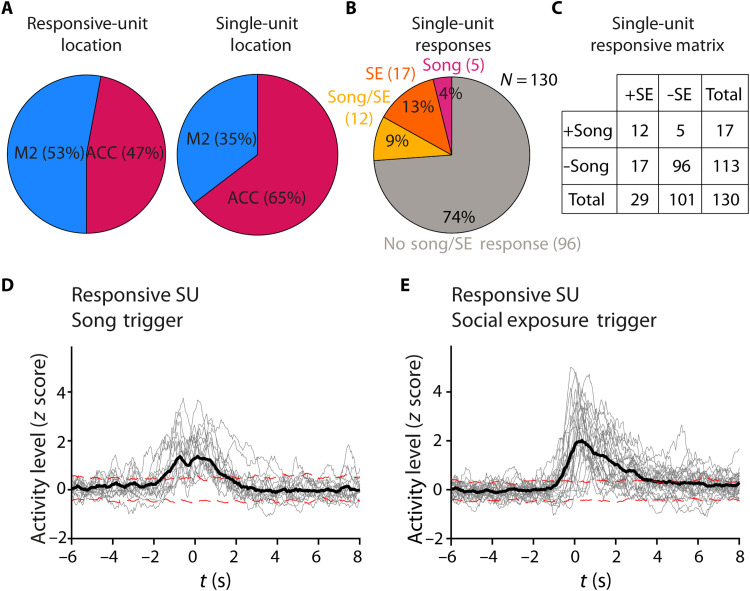
Two overlapping populations of neurons modulate their activity preceding song initiation and SE. (**A**) Basic response properties across all single units. Most of the single units recorded were in ACC, yet of the 34 (22%) responsive single units, only 50% were in ACC. (**B**) 25% of neurons recorded were responding to either song or SE. (**C**) The responsive matrix lists the number in each group. +Song represents the neurons responsive to song, while +SE represents neurons responsive to SE. These categories do not indicate the absence of one of the groups (i.e., −SE only indicates lack of response, not lack of SEs in that group). Timing of responses was also not considered for this classification. (**D**) Averaging all 34 responsive single units on SE reveals a strong response centered around 1 s after SE in the PETH as compared to ±2 SD of a permutation test. This response is stronger than when averaging on song initiation. By separating trials when song or quiet follows SE, we can explore a USV modulatory effect. (**E**) For 17 song responsive neurons, averaging the PETH on song initiation yielded a significant increase in activity preceding song initiation as compared to the dotted red lines representing ±2 SD in a permutation test. However, most song initiations were preceded by SEs, limiting the scope of this result.

To quantify the modulation of an individual unit’s activity with respect to SE and song initiation, we selected units if their activity deviated by two or more SDs compared to baseline (determined by a permutation test). We found 34 units that passed this test and showed a significant change in firing rate with respect to either SE or song initiation. Both M2 and ACC had equal amounts of responsive neurons (Fig. 6A).

The average PETHs of SE-responsive (SE+) single units show an elevation in firing rate when compared to a permutation test (Fig. 6, B and C). The PETH with respect to song initiation (S+) reveals song-related modulatory activity in half (17 of 34) of responding units. These average responses are seen regardless of individual peak response time, as units respond with jittery peak times (note individual traces in Fig. 6, D and E). Thus, the average song PETH shows a significant firing rate elevation beginning at 1.6 s before the song (Fig. 6D). About one-third of the units (36%, 12 of 34) show a significant elevation in firing rate when triggered on either SE or song initiation (examples in Fig. 5). In our experimental paradigm, it was impossible to determine the exact moment of SE and, therefore, difficult to draw conclusions about the fine temporal relationship between SE and the change in firing rate. Nevertheless, we can conclude that 85% (29 of 34) of responding neurons have demonstrated a significant elevation in firing rate in response to SE (Fig. 6E).

**Fig. 7.**
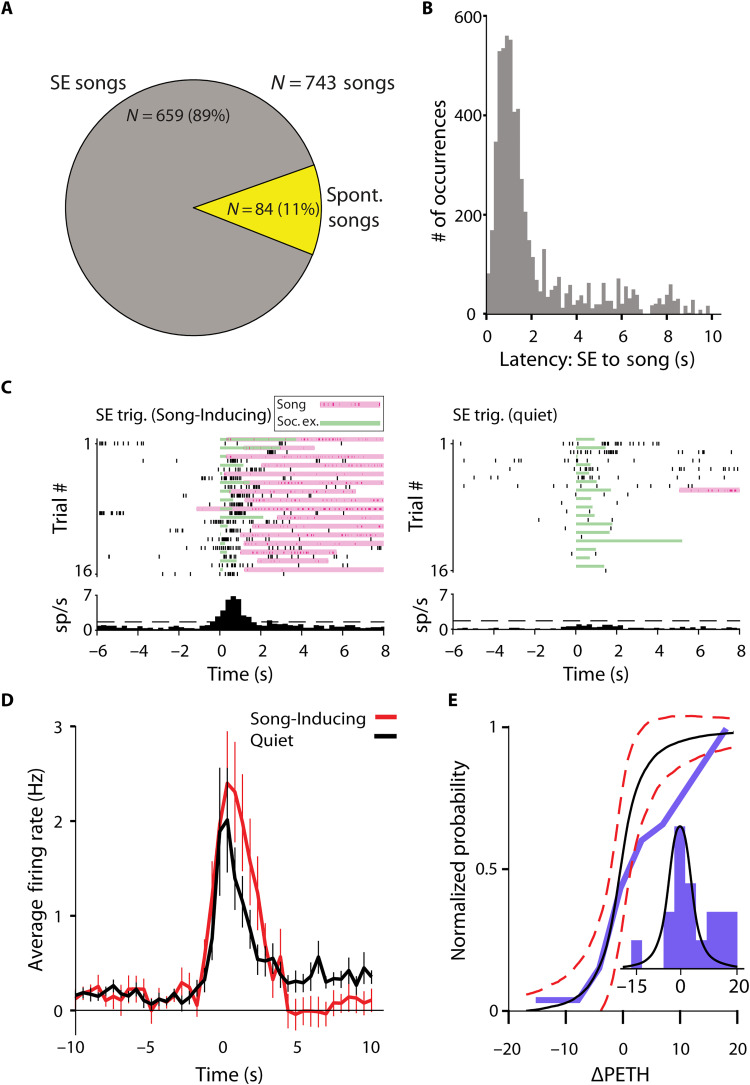
Conditioning on SE reveals an USV modulatory effect on ACC and secondary motor area neural activity. (**A**) Most songs are preceded by SE. In 89% of song emission, mice were exposed to a female, while only 11% of songs were initiated in isolation (at least 5 s of isolation from female SE). (**B**) Most songs occur ~1 to 2 s following SE. Thus, SE is a confound when averaging neural activity triggered on song initiation. (**C**) Two example PETHs from a single unit showing responses triggered on SE, separated to SE resulting in a song or not (quiet). In the above PETH, a Song-Inducing SE reveals an increase in activity about 1 s after exposure. On the other hand, a Quiet SE shows no increase in activity. (**D**) Average SE triggered PETH across all responding neurons (*N* = 18, maximum 40 SEs per neuron) split between Song-Inducing (red) and Quiet (black) SEs. Song-Inducing SEs have a higher firing rate following exposure and a subsequent inhibition. For each unit, the SE-triggered PETH for Song-Inducing and Quiet conditions were calculated (fig. S5) and the difference between them was measured as ΔPETH. (**E**) The cumulative distribution of ΔPETH is shown (blue). The black line (predicted) is calculated by a permutation test. One thousand randomly shuffled groupings of the Quiet/Song-Inducing signal were chosen and the ΔPETH was averaged. The ΔPETH distribution (blue) is shifted to the right compared to the random grouping, representing the increase in single units preferring Song-Inducing SEs.

Figure 6 suggests that the average firing rate of some units in the ACC and in M2 is increased before song initiation. However, it is difficult to determine whether the elevation indeed reflects involvement in song emission because the song initiations were almost always preceded by SE to a female stimulus. In this experiment, 89% of song initiations were preceded by an SE (Fig. 7A). Only 11% of songs were initiated in isolation. Therefore, it is possible that the elevation in firing rate seen in the PETH of S+ units merely reflects a response to SE. We examined the latency from SE to vocal production and found that most songs occur within 2 s of SE (Fig. 7B); therefore, it is impossible to separate the two responses fully.

However, not every SE resulted in song emission [1079 of 1661 SEs resulted in songs (65%)]. Therefore, we have computed the SE-triggered PETH separately for SEs that were followed by a song and for the SE followed by silence. Figure 7C depicts PETHs of one SE+ single unit, separating out the trials with songs or silence following SE. During SEs followed by song production, as opposed to quiet, this unit shows an increase in activity (example in movies S7 and S8). Of 34 responding units, we selected 18 neurons with an average firing rate greater than 1 Hz during SE for this analysis. We further categorize their responses as Song-Inducing (10 of 18), Quiet (3 of 18), or Agnostic (5 of 18) single units (the individual PETH of all units are depicted in fig. S5).

The figure shows that most of the SE+ units modulate their SE-triggered activity depending on whether the mouse will produce a song or remain quiet. Reanalyzing data from the photometry experiment also confirms this Song/Quiet SE modulation (fig. S4, D and E). Therefore, even considering the confound of song-triggered PETHs, which could reflect SE response, cortical neurons modulate their activity with respect to song initiation and their modulation has predictive power. By averaging all SE-triggered PETHs separately for song trials and quiet trials, we found that in sum, neurons positively modulate their firing rate in response to Song-Inducing SE (Fig. 7D and see also fig. S5). In agreement with the average elevation in firing rate, analysis of the responses of individual neurons shows the same tendency. We quantified this by analyzing the distribution of individual cells using the difference between the PETH in the Song-Inducing and Quiet conditions (ΔPETH) across all responsive single units. We found that the real distribution of ΔPETH is shifted toward single units responding more toward Song-Inducing preference in comparison to a shuffled distribution (Fig. 7E). Thus, vocal production modulates the SE response of ACC and M2 neurons.

## DISCUSSION

We demonstrated that the cortex plays a role in the male mouse USV courtship behavior. Intracortical microstimulation in a restricted area of the ACC was sufficient to invoke USVs in head-restrained mice that were absolutely mute otherwise. This was achieved in male mice who never encountered a female mouse or received any other stimulation such as female mouse urine or other odorants. Furthermore, successful trials, in which USVs were elicited, occurred only with long-duration stimulation in a restricted area of the ACC and not in other candidate cortical areas. Bilateral optogenetic stimulation of ACC CamkII-positive neurons of freely exploring males reliably elicited full USV sequences similar to female-induced vocalizations, further supporting the ICMS conclusion, and eliminating the possibility that ICMS-induced USVs resulted from activating passing axons instead of cell bodies. Using fiber photometry, we identified Ca^2+^ signals from the same ACC location that preceded song initiation. We also found that when neural activity in the ACC is optogenetically suppressed, mice produce fewer syllables and initiate fewer songs in the presence of female mice. These results elucidated a possible function for ACC in initiating songs and propose the ACC as an important node in the USV-controlling network. Last, using electrophysiology, we have shown that units in M2 and the ACC respond with firing rate elevation to female exposure and before song initiation. Moreover, many units respond to female exposure differently depending on the future vocal response, whether that response is a song or a quiet period.

Our results fit well with the literature on the involvement of the cortex in vocalizations in many mammals such as monkey, rat, and bat. Purposeful, but not spontaneous, vocal behavior in monkeys is associated with increased ACC activity (*46*). In bats, prefrontal neurons fire differentially in response to calls from different individuals in a social setting (*21*). Previous ICMS and local field potential recording studies in rats demonstrated a role for the ACC in USV production (*26*, *47*, *48*). Rats produce a type of 50-kHz USV, which is induced spontaneously or under a variety of conditions from handling to feeding (*49*). Tickling or ICMS of somatosensory areas also evokes this USV behavior (*50*–*52*). Rat USVs may be a general expression of emotional state, whereas mouse USVs are likely directed toward certain well-defined behaviors as in courtship or other social contexts. In a strain of wild mice, vocalizations arise in a unique behavioral context, and stimulation in the motor cortex disrupts vocalization timing (*23*). In subcortical areas, recent results reveal the direct role of the POA, PAG, and nucleus ambiguus in USV production (*11*–*14*). The short latency and greater reliability of evoked USVs in those studies, as compared to USVs triggered by ICMS or optogenetics in the ACC, demonstrate the direct connection between subcortical brain areas and the motor control of USVs. However, the role of the ACC in affecting these areas has not been studied in the mouse. Moreover, the long latency and the unreliability in inducing USVs could also be a result of the tonic inhibition present in subcortical regions controlling USVs (*11*, *12*).

Most of our experiments were performed on the left ACC mostly for technical reasons. Hemispheric dominance across species has been noted in relation to vocalizations (*53*). In this case, however, upon bilateral optogenetic stimulation of ACC neurons, mice robustly emitted full USV sequences. When stimulated unilaterally in the right or left hemisphere, mice only produced at most a few syllables in an entire session. It would be of great interest to further understand how each side of ACC contributes to vocal production by using, for example, bilateral simultaneous Neuropixels electrophysiology recordings.

Our results challenge previous studies focusing on the anatomical aspects of courtship USVs. A previous finding showed that the motor cortex in mice has a direct monosynaptic connection to the nucleus ambiguus, where laryngeal motor neurons reside (*22*). That finding also showed vocalization-driven immediate early gene activation in M1 and M2. However, our electrical stimulation in the motor cortex, while successful in eliciting complex motor responses, failed to elicit any vocal response. These two results could still be consistent if, for example, other areas are continuously inhibiting vocal production (*11*, *12*). Previous work revealed that POA inhibition onto PAG inhibitory interneurons evokes USV production (*11*, *12*). However, this putative PAG “gate” must either spontaneously fire or receive input to trigger USVs when inhibition is removed. Alternatively, vocal production could require coordination of several processes, and the motor cortex might only exert partial control. In contrast to our motor cortex ICMS, our ACC ICMS successfully triggered USVs. This suggests that the ACC resides higher in the control hierarchy of vocal control. When the cortex (including the ACC) is removed through genetic manipulation, mice still vocalize (*24*). Because the analysis showed no statistical differences between the USVs produced by control and mutant mice, the authors concluded that the cortex is not necessary for USV production and control. However, recent work from the same authors has instead shown that a more sophisticated analysis can separate USVs produced by controls and mutants (*25*). A more sophisticated analysis of the ACC suppression experiment may reveal differences in fine-grained social behaviors that may be correlated with vocal behavior. Therefore, our results are in contrast to the earlier finding but are consistent with the latter and with the view that the cerebral cortex is likely to exert complex control over innate behaviors.

Courtship-related USV behavior is a complex behavior with many concurrent and related subbehaviors. We separated responses to SE on the basis of whether mice vocalized or remained quiet, in an attempt to understand the unique contribution neural activity in the ACC has on vocal behavior. However, the role of ACC activity in vocal behavior is likely indirect and may control certain higher-level features of vocal behavior that elude a simple quantification. Future studies can target more specific cell populations bilaterally in the freely moving mouse to further understand the exact role of ACC in vocal behavior. We do not account for social or sexual motivation as another factor in ACC activity; however, these factors usually have longer timescales than the few seconds of increased ACC activity as seen in the electrophysiology recordings. Our study is a step toward understanding the role of cortex in mouse vocal behavior.

The involvement of the ACC in the mouse USV process opens the option that this process is more complex and plastic than currently assumed. It is already known that mice vocalize differently when social context is varied (*1*, *54*–*56*) and that one study found that mice can modulate their pitch in a cross-strain fostering scenario; however, other studies question this finding (*8*, *10*, *57*). The access in the mouse to specific cell populations, retrograde labeling, and genetic manipulations provides a unique opportunity to explore and manipulate higher-order control on the USV-controlling network and ultimately relate it to human speech in health and in various forms of neurological and genetic conditions in which it is disrupted (*58*–*61*, *61*–*67*).

## MATERIALS AND METHODS

### Animals

We used C57BL/6 male mice unless otherwise noted (at least 8 weeks old). In addition, we used several genetically modified mouse lines (Camk2, Rbp4, Thy1, Rbp4-Ai32, and Pv-CRE): (B6.Cg-Tg(Camk2a-cre)T29-1Stl/J from the Jackson Laboratory, Tg(Rbp4-cre) from the Jackson Laboratory, B6.Cg-Tg(Thy1-COP4/EYFP)18Gfng/J from the Jackson Laboratory, B6;129S-Gt(ROSA)26Sortm32(CAG-COP4*H134R/EYFP)Hze/J from the Jackson Laboratory crossed with RBP4 mice as above, and B6;129P2-Pvalbtm1(cre)Arbr/J from the Jackson Laboratory. The Hebrew University Animal Care and Use Committee approved all experiments (IACUC NS-16-14216-3, “Functional identification of brain regions involved in ultrasonic vocalization in mice.”).

### Surgery and viral vectors injections

During surgery, mice were anesthetized with isoflurane (1 to 2% by volume in O_2_, LEI Medical). Rymadyl analgesia (10 mg/kg body weight, 200-μl injection volume) was administered before incision. Mice were put in a stereotactic frame (Narishige, Japan/Luigs & Neumann GmBH), and a small craniotomy <1 mm in diameter was made over the left ACC (dura was not removed). Virus-containing solution was slowly injected (1 to 2 nl/s) through a quartz pipette (pulled on P-2000, Sutter Instrument) using a Nanoject 3 system (Drummond Scientific Company, PA) (500 nl, one site per animal at a depth of 1400 to 1700 um). After injection, the craniotomy was sealed with bone wax, and the skin was attached with VetBond (3 M). Two to 3 weeks after the virus injection, the mice were prepared for optical stimulation. Surgery was performed under isoflurane anesthesia, and Rymadyl analgesia was given. For the optical stimulation experiments, a custom-made fiber optical cannula was subsequently implanted over the injection site. For animals undergoing fiber-photometry experiments, an optical fiber was directly connected to the skull with dental cement under the same conditions. For ICMS experiments, a head post was fixed on the skull under the same surgical conditions and subsequently awakened and allowed to rest for 2 to 3 days before ICMS began. For electrophysiology recordings, a head post and ground pin were fixed on the skull under the surgical conditions and subsequently awakened and allowed to rest for 2 to 3 days before recording sessions began.

### Sound recording and USV analysis

In all experiments, sound was recorded to capture USVs using an ultrasonic microphone (Avisoft, CM16/CMPA) amplified (either by CMPA40-5 V or UltraSoundGate 116H) and digitized at 250 kHz (either by National Instruments Card or Avisoft RECORDER). In fiber-photometry and optogenetic experiments, males were isolated for 5 min in a new cage and presented with a female either by adding it to the same cage or by presenting it on the experimenter’s hand. In electrophysiology experiments, head-restrained males were presented with a female mouse or female mouse urine only on the experimenter’s hand to induce vocal production. We parsed USVs using an algorithm previously published available at https://github.com/london-lab/MouseUSVs/tree/master/USV_Parsing (*16*). In brief, the raw audio signal is cleaned for common noise sources, and pixels in the spectrogram are removed if they are isolated from other pixels. For the algorithm parameters, we used a frequency cutoff between 20 and 125 kHz and a square size of 3. In all subsequent analysis, a syllable is defined as a continuous frequency modulation in the spectrogram of at least 8 ms in duration with a surrounding silent period of at least 16 ms. In addition, a song is defined as a series of syllables with a surrounding silent period of at least 10 s. We determined syllable features using the following methods: Duration was determined by subtracting the end from the start time of the syllable as defined using our parsing algorithm. Mean frequency was determined by using another threshold on the syllable spectrogram to clean noise and then taking the average of all remaining pixels across frequency. Frequency bandwidth was determined on the same spectrogram by subtracting the highest frequency from the lowest frequency remaining in the spectrogram.

### Intracortical electrical microstimulation

Intracortical microstimulation was performed on head-restrained awake male mice to map cortical coordinates to complex behaviors (*26*, *32*, *65*, *66*). All mice were group housed and naïve to females after weaning at P21. A broken pipette tip was lowered into the brain with a micromanipulator. Each site was stimulated between 10 and 100 times with a current stimulator of World Precision Instruments (FL). The stimuli consisted of either a 50-ms (short) or 200- to 500-ms (long) duration train of 333-Hz stimulation varying between positive and negative currents (biphasic square wave) between 10 and 100 μA. The large stimulus amplitudes are similar to other ICMS studies looking at complex behaviors (*24*, *33*).

### Optical stimulation

Channelrhodopsin 2 (ChR2) was injected bilaterally as described on a CamKII promotor in the AAV9 viral serotype into transgenic mice expressing CRE in CamkII-positive neurons [B6.Cg-Tg(Camk2a-cre)T29-1Stl/J from the Jackson Laboratory]. Fiber optical cannulas (multimode fibers FT-200-URT, Thorlabs, Grünberg, Germany) with a diameter of 200 μm and a NA of 0.22 were implanted at a 10° angle into ACC at coordinates (700 lateral, 700 anterior) and covered in dental cement. After recovery and 2 weeks to allow for viral expression, mice were tested while exploring either their home cage or a fresh cage with no female present before or during the session. Light was delivered through a 100-mW laser (1.5 to 3 mW/mm^2^ using a 473-nm laser; Changchun New Industries Optoelectronics, Changchun, China) attached to a rotary joint splitter (Part # RJ2, Thorlabs, Grünberg, Germany) to allow for free exploration. ACC neurons were activated with 10 s of stimulation with about 30 s of rest between stimulations. Stimulus trains consisted of 20-Hz pulses of 10 ms.

### Fiber photometry

We used multimode fibers (FT-200-URT, Thorlabs, Grünberg, Germany) with a diameter of 200 μm and an NA of 0.22. After removing the cladding from the fiber tip, the tip of the fiber was inserted into the brain and subsequently covered with dental cement. Low-intensity stimulation (typically <0.1 mW at the tip of the optical fiber) was delivered by a 20-mW solid-state laser (Sapphire, 488 nm, Coherent, Dieburg, Germany) for excitation of the GCaMP6s indicator and recording of Ca^2+^-dependent fluorescence was collected through the same fiber to an avalanche photodiode (APD, S5343, Hamamatsu Photonics, Herrsching, Germany). The resulting voltage signal was oversampled at 50 kHz and stored for offline analysis.

### Optical supression

GtACR2 was injected as described on a CamKII promotor in the AAV5 viral serotype (*41*). Fiber optical cannulas were implanted as above into ACC. After recovery, male mice interacted with female mice in a prelight baseline session. ACC neurons were suppressed in the second session after 2 days by optical stimuli from laser illumination (1.5 to 3 mW/mm^2^ using a 473-nm laser; Changchun New Industries Optoelectronics, Changchun, China). Fifty stimuli at 20 Hz for a duration of 10 s were given semirandomly, ensuring that about every 4 min, at least 10 stimuli occurred. Short pulses of stimuli were given before the session began to calibrate the laser power to below the threshold for movement initiation.

### ICMS, fiber photometry, and optical stimulation data analysis

All analysis was conducted offline using custom code written in MATLAB 9.9 (MathWorks). In ICMS experiments, each audio file was manually reviewed at least twice to identify any USVs. In fiber-photometry experiments, relative fluorescence changes Δ*f*/*f* = (*f* − *f*0)/*f*0 were calculated as Ca^2+^ signals relative to baseline, where the baseline fluorescence was determined as the 10th percentile of the signal. For optical stimulation and fiber-photometry experiments, the USV parsing was as described above.

### Neuropixels recording and analysis

The Neuropixels probe is a high-density and high signal-to-noise extracellular probe [Jun *et al.* (*45*)]. The probe was soldered to short the reference pad to ground, and ground was connected to a pin in the head post. The probe was held on a custom metal-bar attached to a Luigs & Neumann (Ratingen, Germany) micromanipulator. Before recording, mice were habituated to head restraint using their home-cage running wheel in the experimental setup. Females were briefly presented to head-restrained males to induce vocal behavior. We call these presentations “social exposures,” which ended shortly after the experimenter saw that the mice started to vocalize. This was done to maximize vocal output in the short period that mice can be head restrained. SEs that did not evoke vocalizations did not go beyond 10 s, as female mice were a constant danger of breaking the Neuropixels probe. A brief craniotomy surgery was performed as described above to achieve access to cortex. The craniotomy was continuously filled with artificial cerebrospinal fluid and sealed between experiments. We recorded signals in 11 experiments from three mice in the left hemisphere ACC and M2 at a maximum depth between 2000 and 2500 μm. Probes were allowed to settle for about 10 min, while probe gain calibration occurred. All recordings were made using SpikeGLX software. All data were preprocessed using the common average reference by subtracting the common median across all channels [Ludwig *et al.* (*68*)]. Single-unit channels were selected by manual inspection. In the case that a single unit was on multiple channels, the channel with the largest spike waveform was chosen. Subsequently, spikes were detected as threshold crossings greater than 10 SDs from the mean.

### Video analysis for Neuropixels electrophysiology

Video was recorded at 30 frames per second concurrently with Neuropixels and sound data using a blue light-emitting diode as a synchronization signal. Off-line, the presence of females was extracted by using a region of interest in front of the head-restrained mouse snout to automatically detect changes in pixel strength to indicate a possible behavioral event. Subsequently, each possible behavioral event was manually confirmed with start and end frames determining SE times.

### Statistical analysis

Statistical significance was assessed using the unpaired Student’s two-sided *t* test unless otherwise specified. When comparing USV properties, paired Student’s two-sided *t* test was used between animals for each property. Significant level was marked as *P < 0.05, **P < 0.01, and ***P < 0.001. All data were reported as means ± SEM unless otherwise specified.

### Viral vector list

Viral vectors are as follows:

AAV9-CAG:: GCaMP6S.WPRE.SV40 (Penn Vector Core, titer 3.28 × 10^13^, 500 nl per site, LOT CS0775)

AAV9-CAG-FLEX-rev-Chr2-TdTomato (ELSC Vector Core facility, titer 3 × 10^13^, 300 nl per site)

AAV5-CK2:: GtACR2.Fred.kv.2.1 (ELSC Vector Core facility, titer 1 × 10^11^, 500 nl per site)

AAVdj-CMV:: eGFP (ELSC Vector Core facility, titer 5 × 10^12^, 300 nl per site)

AAV9-CAG-FLEX-TdTomato (ELSC Vector Core facility, titer 5 × 10^12^, 300 nl per site).
