# Supplementary material for: Cortical circuits modulate mouse social vocalizations
Source: Sci Adv. 2023 Sep 29;9(39):eade6992. doi: 10.1126/sciadv.ade6992 (PMC10541007; doi:10.1126/sciadv.ade6992)
Supplement: Supplementary file 1 — Figs. S1 to S5 Table S1 Legends for movies S1 to S8 Legend for data S1 [file sciadv.ade6992_sm.pdf]

Science Advances
AAAS

Supplementary Materials for

Cortical circuits modulate mouse social vocalizations

Benjamin Gan-Or and Michael London

Corresponding author: Michael London, mickey.london@mail.huji.ac.il

Sci. Adv. **9**, eade6992 (2023)
DOI: 10.1126/sciadv.ade6992

The PDF file includes:

Figs. S1 to S5
Tables S1
Legends for movies S1 to S8
Legend for data S1

Other Supplementary Material for this manuscript includes the following:

Movies S1 to S8
Data S1

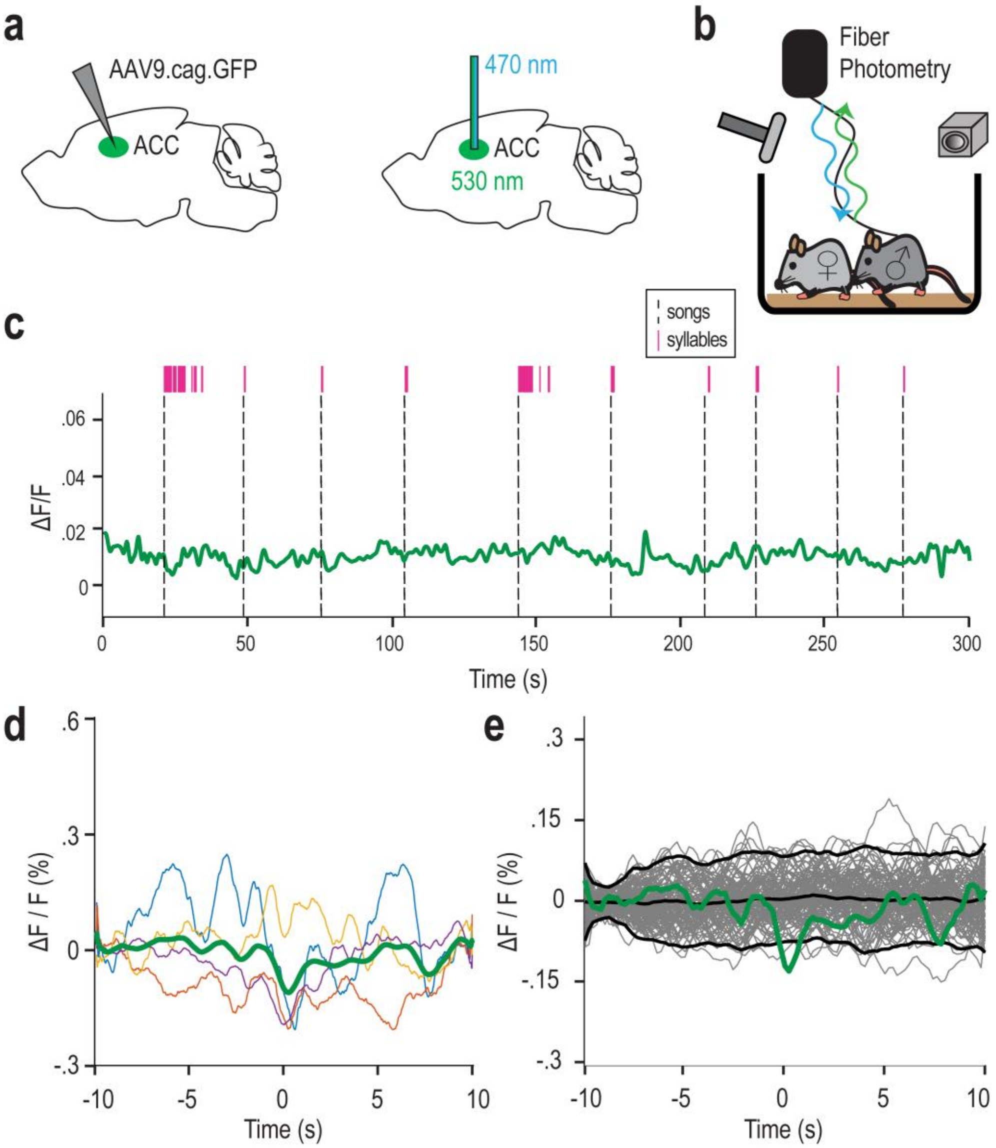

Fig. S1.

Accompanying Fig. 3. GFP activity in the ACC remains constant during song initiation. A. Virus with GFP fluorescent indicator was injected two to three weeks before recording. Recordings were made from the left ACC using blue light to activate the GFP protein and green light was detected in the photodiode. B. Schematic illustration of fiber photometry setup. Male mice with fiber implants were allowed to move freely in a cage, while a female was introduced to elicit USVs. C. Example session of 5 minutes in duration. The green line represents GFP signal, thin magenta lines are syllables, and the dotted black line represents song initiations. D. Average GFP signals for all mice, triggered on the first syllable in a song separated by at least 10 seconds. E: Permutation test trials using random trial times plotted in gray. The average from D in green is plotted against the average and ±2 SD of the permutation test average in black. The response is mostly within the noise range of the permutation test, with a small but weakly significant decrease in activity during vocal onset.

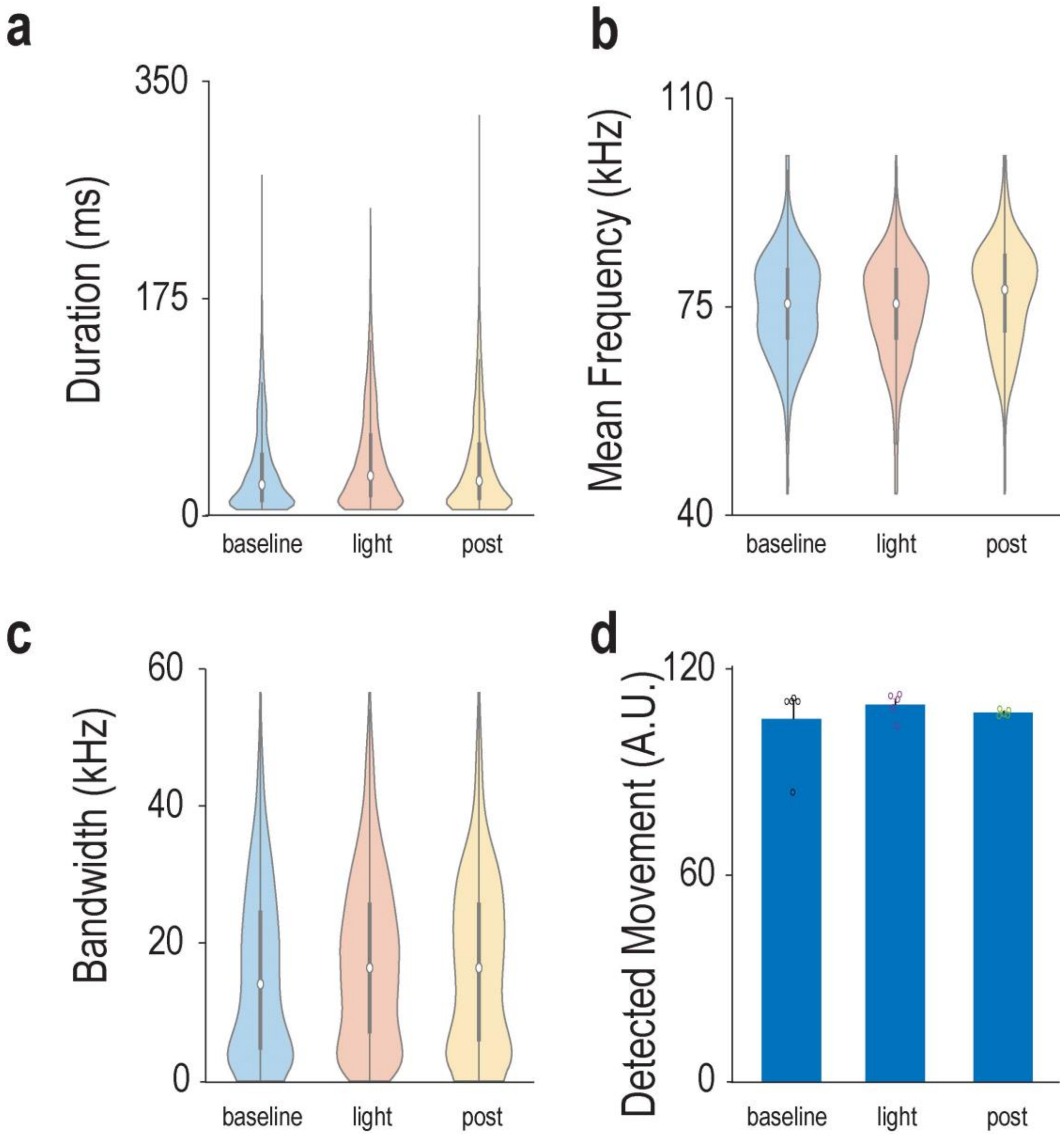

Fig. S2.

Accompanying Fig. 4. A-C. Comparing vocalization acoustic features across sessions. Acoustic features remained qualitatively similar between sessions as syllable and song rates were reduced in the light session. When comparing animals, there was no significant difference between features (n=5 mice, paired t-tests: Duration, baseline[43.4±0.7ms]/light[41.4±3.5ms] p=0.60 or light[41.4±3.5ms]/post[42.1±0.3ms] p=0.86; Mean frequency, baseline [74.7±0.9 kHz]/light [74.6±1.6 kHz] p=0.93 or light [74.6±1.6 kHz]/post [76.3±1 kHz] p= 0.43; Bandwidth, baseline [17.9±0.8 kHz]/light [16.2±2.7 kHz] p= 0.56 or light [16.2±2.7 kHz]/post [16.7±0.9 kHz] p=0.86) B. A comparison of movement using pixel change detection. The number of pixels between frames was counted and smoothed and averaged across sessions and animals. No significant difference was found between sessions, indicating that ACC suppression does not affect the locomotor activity of mice.

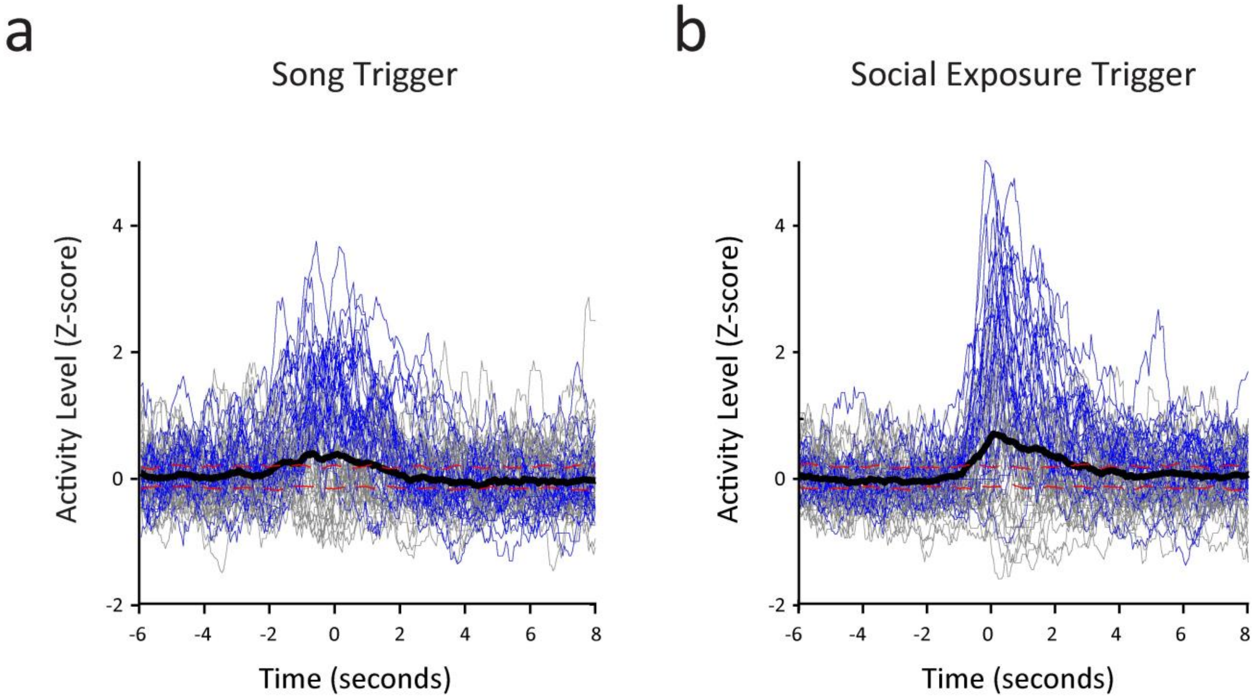

Fig. S3.

Accompanying Fig. 5. Averaging over all responsive neurons reveals a social exposure and song initiation response. A. Averaging all 130 responsive single units (black line) on social exposure. The result is similar, but weaker, than seen with only responsive neurons. Red dotted lines represent ±2 SD of a permutation test, while gray lines represent individual single units. Blue lines represent responding units (34/130). B. Same, but averaging at time zero on song initiations.

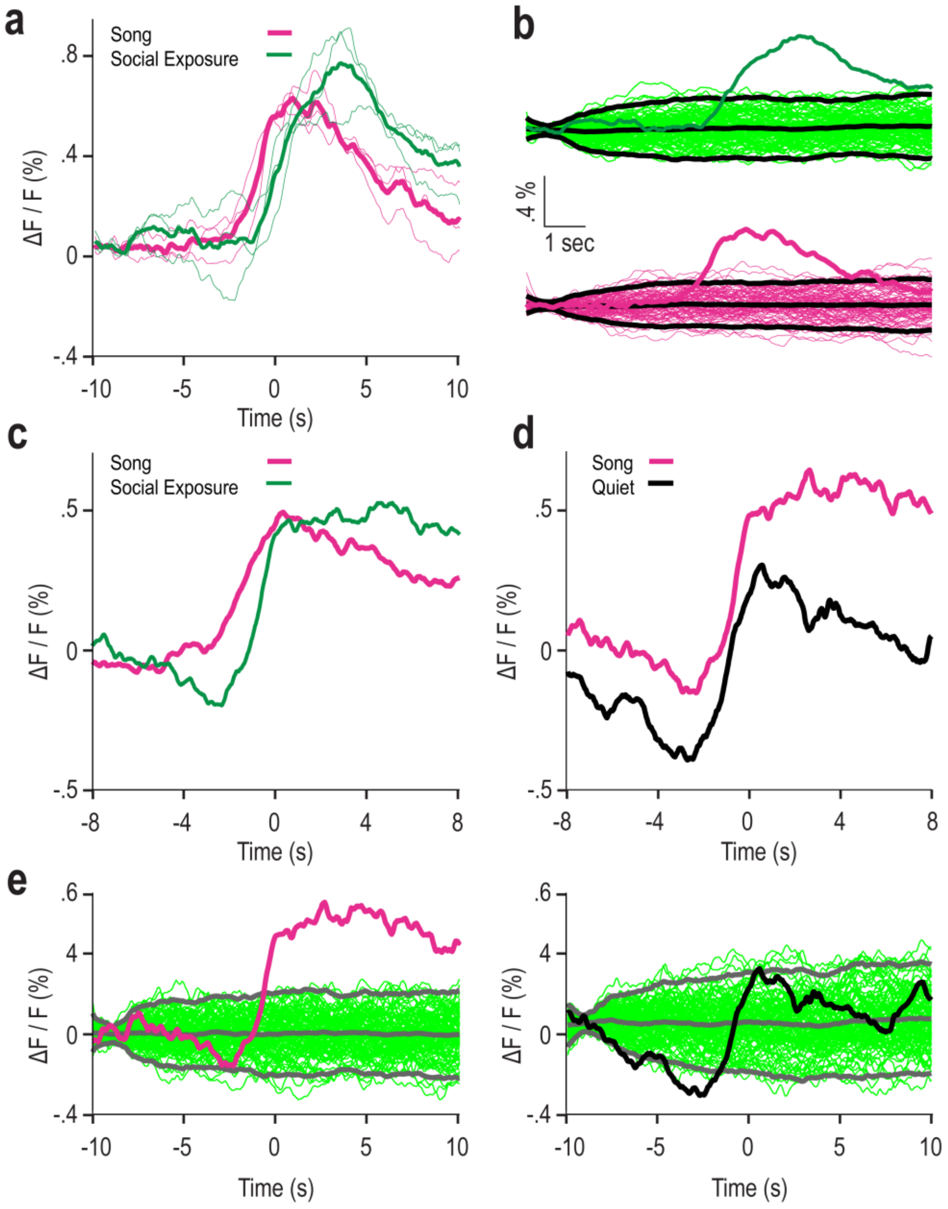

Fig. S4.

Accompanying data from Fig. 3. Anterior Cingulate Cortex Ca2+ activity separated triggering on the song or social exposure in 3/5 mice. A. Average Ca2+ signals for selected mice, triggered on the first syllable in a song in magenta and the social exposure time as determined by manual video analysis in green. B. Average activity of both triggers with corresponding permutation tests. Both triggers show significant increases with the social exposure triggered average showing a delayed response compared to song, as noted in Fig. 7B. C: In a single mouse, social exposure trials could be compared as resulting in either song or in quiet. This separation shows either the song or social exposure triggers for that mouse. D. Splitting up the social exposure triggers into song or quiet reveals a similar result as seen in Fig. 7 with electrophysiology data. E: Permutation test trials using random trial times plotted in light green. The averages from D are in magenta representing song or black representing quiet trials. Thick black lines are the average and ±2 SD of the permutation test. Note that social exposure triggered averages resulting in song is highly significant, whereas SE trials averaged resulting in quiet is not.

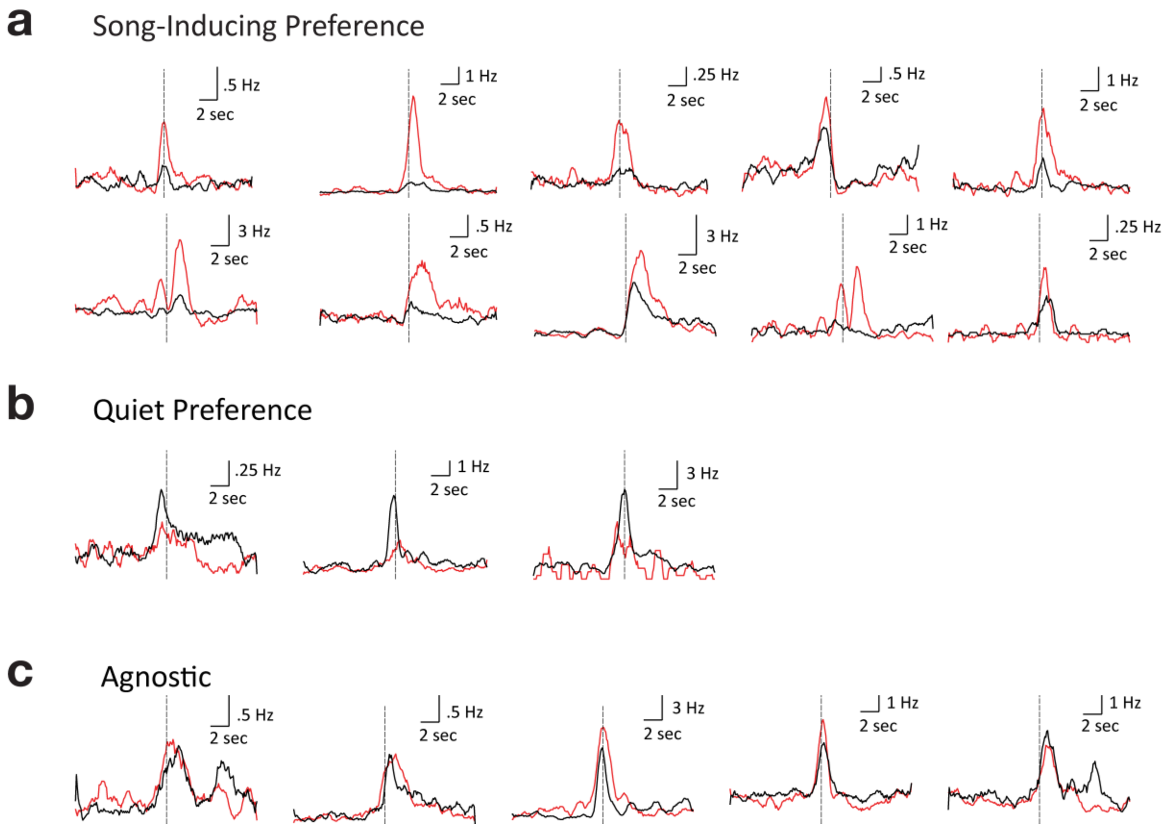

Fig. S5.

Accompanying Fig. 7. Single units were categorized by their response to social exposure into 3 groups: A. Song-Inducing, B. Quiet, or C. Agnostic. Most single units prefer the Song-Inducing category. Each dotted line represents a social exposure with 10 seconds of activity before and after.

Duration(ms)	30.50 +- 23.62
Freq Start (kHz)	84.17 +- 15.75
Freq Mean (kHz)	81.08 +- 14.08
Freq Max (kHz)	90.83 +- 16.86
Freq Bandwidth (kHz)	18.18 +- 14.34

Table S1.

Microstimulation ultrasonic vocalization parameters

Movie S1.

Representative example of freely moving USV behavior. Left frame is video recorded from above cage. Right frame is spectrogram representation of sound. Sound in video is processed using a vocoder to hear in the human frequency range.

Movie S2.

Representative example of head-restrained USV behavior. Left frame is video recorded from above cage. Right frame is spectrogram representation of sound. Sound in video is processed using a vocoder to hear in the human frequency range.

Movie S3.

Accompanying Fig. 1, an example of ICMS-induced USV behavior. Left frame is video recorded from above cage. Right frame is spectrogram representation of sound. Sound in video is processed using a vocoder to hear in the human frequency range.

Movie S4.

Accompanying Fig. 2, an example of three optogenetic activations in ACC. Left frame is video recorded from above cage. Right frame is spectrogram representation of sound. Sound in video is processed using a vocoder to hear in the human frequency range. Part of the rest time between stimulations are cut off for brevity.

Movie S5.

Accompanying Fig. 3, an example of fiber photometry recording setup in ACC and evoked Ca^{2+} signal. Left frame is video recorded from above cage. Right frame is spectrogram representation of sound above with Ca^{2+} signal below. Sound in video is processed using a vocoder to hear in the human frequency range.

Movie S6.

Accompanying Fig. 4, an example of optogenetic suppression in ACC. Left frame is video recorded from above cage. Right frame is spectrogram representation of sound. Sound in video is processed using a vocoder to hear in the human frequency range.

Movie S7.

Accompanying Fig. 7, an example of Neuropixels recording from 14 single units during a social exposure followed by song production. Left frame is video recorded from above cage. Right frame is spectrogram representation of sound above and black lines below representing spikes above threshold. Sound in video is processed using a vocoder to hear in the human frequency range. One single unit's spike activity was interposed with the audio.

Movie S8.

Accompanying Fig. 7, an example of Neuropixels recording from 14 single units during a social exposure followed by quiet. Left frame is video recorded from above cage. Right frame is spectrogram representation of sound above and black lines below representing spikes above threshold. Sound in video is processed using a vocoder to hear in the human frequency range. One single unit's spike activity was interposed with the audio.

Data S1.

Excel Sheet 1. ICMS evoked USV latency. Panel D shows histogram data of latency from stimulation onset to USV onset.

Excel Sheet 2. Optogenetically evoked USVs. Panel D (left) shows histogram data of latency from stimulation offset to USV onset. Panel D (right) shows the syllable rate for each individual mouse in the time window after stimulation offset. Panel E details all of the experimental paradigms attempted and the success in # of evoked syllables. Panel F compares three vocalization properties: duration, mean frequency, and bandwidth between female and optogenetically induced syllables.

Excel Sheet 3. Fiber photometry recordings during natural vocal behavior. Panel D shows average GcAMP signal triggered on vocal initiation for each mouse across time. Time zero represents the vocal initiation. Panel E compares the real data average to an average obtained by using random trigger times.

Excel Sheet 4. Optogenetically suppressed USVs. Panel D and E quantifies the differences between syllables and song initiations between baseline, light, and post sessions in the experiment.

Excel Sheet 5. Neuropixels electrophysiology during social vocalization examples. Panel C through F show example single units triggered on the left by song initiation and on the right by social exposure. Each post-event time histogram (PETH) is centered on the respective trigger and shows spikes per second.

Excel Sheet 6. Neuropixels electrophysiology averages. Panel D and E show the averaged responses of all responsive single units to song initiation and social exposure triggers, respectively. Upper and lower bounds are determined by random triggers.

Excel Sheet 7. Neuropixels electrophysiology comparing social exposure triggers leading to song or quiet. Panel B demonstrates the latency from social exposure to song initiation. Panel C is composed of two PETHs, on the left with song-inducing trials and the right quiet trials. Panel D is an average of the difference between song-inducing and quiet SU responses with standard error bars. Panel E is a quantification of the expected cumulative distribution compared to the actual distribution of single units preferring song-inducing or quiet trials. Predicted and Random lines are determined by assigning random labels to each trial. In the inset, the actual distribution is normalized to fit onto the random distribution.
